# Gastric Xylobezoar Treated with Continuous Enteral Coca-Cola® Infusion

**DOI:** 10.1155/2022/7187356

**Published:** 2022-08-28

**Authors:** Gopinathan Gangadharan Nambiar, Eyad Hanna, Julia Shelton, Rose Lee

**Affiliations:** ^1^Pediatric Gastroenterology Hepatology Pancreatology, Nutrition University of Iowa Stead Family Children's Hospital, Iowa, USA; ^2^Department of Pediatrics, Division of Pediatric Gastroenterology, Hepatology, Pancreatology, Nutrition University of Iowa Stead Family Children's Hospital, Iowa, USA; ^3^Department of Surgery-Pediatric Surgery, University of Iowa Stead Family Children's Hospital, Iowa, USA

## Abstract

Xylobezoar is a rare clinical condition in which undigested paper becomes trapped in the gastrointestinal tract causing varying degrees of gastrointestinal obstruction. This condition can be suspected in children with a history of gastric surgeries, decreased gastrointestinal motility, or pica. Xylobezoar presents with symptoms ranging from chronic abdominal pain to gastrointestinal perforation. Surgical intervention is often required as endoscopic removal is challenging and not always successful. Chemical dissolution has been shown to be effective in treating certain bezoars. Here, we report a case of a pediatric patient with xylobezoar who was successfully treated with continuous enteral Coca-Cola® infusion.

## 1. Introduction

A bezoar is an aggregation of indigestible material that accumulates in the gastrointestinal tract. This indigestible mass can be formed by a variety of intentionally or accidently ingested materials. Bezoars can form and be found in any part of the gastrointestinal tract, though they are mostly found in the stomach [1]. There are different types of bezoars based on composition. These substances can include materials like hair, plant fiber, medications, and milk proteins. Once diagnosed, the bezoar often needs to be endoscopically or surgically removed, as it can cause gastric outlet obstruction, ulcerations due to pressure necrosis, and subsequent gastrointestinal bleeding [2, 3]. Pica, the repeated ingestion of inedible items lacking nutritional value, can lead to the formation of bezoars. This behavior is known to be more prevalent among children with autism spectrum disorder [4]. The overall prevalence of bezoars in children is unknown. Chemical dissolution using various agents like cellulase, papain, and acetylcysteine has shown to be successful in dissolving certain gastric bezoars [5]. Carbonated drinks have been reported to be effective in treating phytobezoars [6]. To the best of our knowledge, there are no previous case reports of xylobezoar treatment using Coca-Cola®.

## 2. Case Report

We report the case of a 14-year-old boy with autism spectrum disorder and pica, who presented to the emergency department with severe abdominal pain and vomiting; emesis was notable for containing paper fragments. The patient has been known to consume paper, pillow foam, dental floss, and other household objects.

The patient also admitted to eating at least two pages of paper daily for the past two weeks. On arrival, he was tachycardic, likely due to pain and dehydration, and his generalized abdominal pain improved with intravenous fluids.

**Figure 1:**
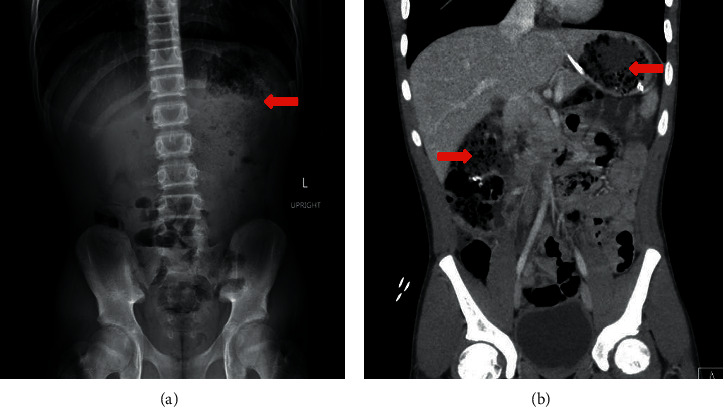
(a) Abdominal X-ray showing large amount of intra-gastric ingested material. (b) CT abdomen (coronal view) showing intra-gastric bezoar without gastric obstruction with similar contents in the proximal small bowel and ascending colon (red arrows).

Upon admission, labs including complete blood count, metabolic panel, amylase, and lipase were unremarkable. Abdominal X-ray showed large amounts of intra-gastric ingested material with a non-obstructive bowel gas pattern. Abdominal CT showed presumed intra-gastric bezoar without gastric outlet obstruction and similar enteric contents in the proximal small bowel and ascending colon (Figure 1).

The differential diagnosis included gastric bezoar, viral gastroenteritis, acute pancreatitis, peptic ulcer disease, and partial small bowel obstruction. History, clinical exam, and workup led to the diagnosis of gastric bezoar.

A nasogastric (NG) tube was placed for gastric decompression. Pediatric surgery was consulted for potential surgical removal. Due to interval improvement in abdominal pain and vomiting, chemical dissolution of the gastric bezoar was attempted. The patient was given two liters of continuous Coca-Cola® infusion via NG tube at the rate of 75 ml/hr over the course of 24 hours with constant monitoring of vital signs and blood glucose levels. The patient tolerated the procedure well without any complications.

**Figure 2:**
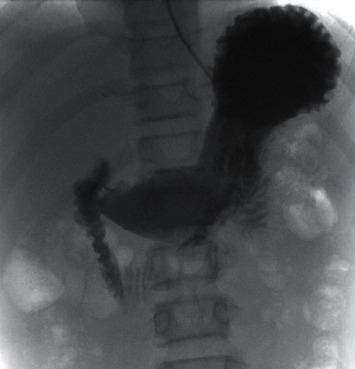
Upper GI study post-Coca-Cola® infusion showing no filling defect in the stomach or small bowel to suggest bezoar.

Following the infusion, an upper GI study was obtained after 12 hours. The study showed no evidence of delayed gastric emptying or small bowel obstruction. There was no filling defect that would suggest a bezoar (Figure 2). The NG tube was removed, and the patient tolerated a diet without emesis or abdominal pain. The patient had a bowel movement containing a large amount of paper prior to discharge.

On follow-up, he denied any gastrointestinal symptoms or adverse effects from chemical dissolution. Behavioral psychology also followed up for the assessment and management of pica. Psychologists recommended various strategies including pica proofing by removing inedible items from the home that are likely to result in pica, identifying edible replacement items, and closer supervision. His pica has improved since hospitalization, and he has expressed a desire to stop this behavior.

## 3. Discussion

Xylobezoar is an aggregation of indigestible paper found anywhere along the gastrointestinal tract. Xylobezoars are rare and only a few cases have been reported in the literature. Our patient is unique as this is the first reported case of successful treatment of xylobezoar using Coca-Cola®.

Xylobezoar can lead to complete gastric outlet obstruction. Even in the absence of gastric outlet obstruction, aggressive treatment is necessary to prevent ulcerations from pressure necrosis and subsequent gastrointestinal bleeding and perforation [1].

The treatment options for a bezoar include dissolution with chemicals, endoscopic retrieval, or surgery [2]. It is often removed surgically because the consistency of the bezoar often causes difficulty for endoscopic fragmentation and retrieval [3].

Chemical dissolution with Coca-Cola® is often used for the initial treatment of uncomplicated gastric bezoars, especially for phytobezoars [5]. The mechanism by which carbonated soft drinks dissolve bezoars is not clearly understood. It is postulated that bezoars are digested by the mucolytic effect of sodium bicarbonate and/or by the acidifying effect of carbonic acid and phosphoric acid while carbon dioxide bubbles penetrate between fibers of the bezoars, thereby increasing the available surface area for the reaction to occur [6].

In our patient, it was unclear whether Coca-Cola® digested the xylobezoar completely or made it more pliable for moving to the small intestine. However, both the gastric and enteric contents were cleared after a slow nasogastric infusion of Coca-Cola® suggesting possible dissolution of the xylobezoar.

Bezoars can mimic various gastrointestinal disorders. Hence, clinicians should consider the possibility of bezoar when evaluating a patient with abdominal pain, a history of pica, and autism spectrum disorder. It is equally important to address the behavioral aspect of the development of bezoars in pediatric patients. A comprehensive treatment plan involving the pediatrician, behavioral psychologist, and caregivers should be implemented for preventing similar life-threatening conditions in the future.

In conclusion, chemical dissolution using Coca-Cola® is a non-invasive option for the treatment of gastric xylobezoar. It can be tried as first-line therapy in a patient without evidence of complete obstruction, bleeding, or perforation in a tertiary care setting with immediate gastroenterology and surgical backup. Partially dissolved bezoars after chemical dissolution can potentially cause small intestinal obstruction, which physicians should be aware of in the treatment of these patients.

## Data Availability

The data supporting the findings of this study are included within the article.
